# Risk Factors for Anastomotic Leakage in Advanced Ovarian Cancer Surgery: A Large Single-Center Experience

**DOI:** 10.1245/s10434-022-11686-y

**Published:** 2022-04-18

**Authors:** Barbara Costantini, Virginia Vargiu, Francesco Santullo, Andrea Rosati, Matteo Bruno, Valerio Gallotta, Claudio Lodoli, Rossana Moroni, Fabio Pacelli, Giovanni Scambia, Anna Fagotti

**Affiliations:** 1 Division of Gynecologic Oncology, Department of Woman and Child Health and Public Health, Fondazione Policlinico Universitario A. Gemelli IRCCS, Università Cattolica del Sacro Cuore, Rome, Italy; 2 Department of Oncology, Gemelli Molise Spa, Campobasso, Italy; 3 Surgical Unit of Peritoneum and Retroperitoneum, Fondazione Policlinico Universitario A. Gemelli IRCCS, Rome, Italy; 4 Fondazione Policlinico Universitario A. Gemelli IRCCS, Rome, Italy; 5 Università Cattolica del Sacro Cuore, Rome, Italy

## Abstract

**Background:**

Cytoreductive surgery is currently the main treatment for advanced epithelial ovarian cancer (OC), and several surgical maneuvers, including colorectal resection, are often needed to achieve no residual disease. High surgical complexity carries an inherent risk of postoperative complications, including anastomosis leakage (AL). Albeit rare, AL is a life-threatening condition. The aim of this single-center retrospective study is to assess the AL rate in patients undergoing colorectal resection and anastomosis during primary surgery for advanced epithelial OC through a standardized surgical technique and to evaluate possible pre/intra- and postoperative risk factors to identify the population at greatest risk.

**Methods:**

A retrospective analysis of clinical and surgical characteristics of 515 patients undergoing colorectal resection and anastomosis during primary or interval debulking surgery between December 2011 and October 2019 was performed. Several pre/intra- and postoperative variables were evaluated by multivariate analysis as potential risk factors for AL.

**Results:**

The overall anastomotic leakage rate was 2.9% (15/515) with a significant negative impact on postoperative course. Body mass index < 18 kg/m^2^, preoperative albumin value lower than 30 mg/dL, section of the inferior mesenteric artery at its origin, and medium–low colorectal anastomosis (< 10 cm from the anal verge) were identified as independent risk factors for AL on multivariate analysis.

**Conclusions:**

AL is confirmed to be an extremely rare but severe postoperative complication of OC surgery, being responsible for increased early postoperative mortality. Preoperative nutritional status and surgical characteristics, such as blood supply and anastomosis level, appear to be the most significant risk factors.

**Supplementary Information:**

The online version contains supplementary material available at 10.1245/s10434-022-11686-y.

Ovarian cancer (OC) is the most fatal of all female reproductive cancers,^1,2^ and given its vague symptoms, it is usually diagnosed at an advanced stage. Complete gross resection (CGR) is the most important prognostic factor, in both primary debulking surgery (PDS)^3^ and interval debulking surgery (IDS);^4,5^ therefore, maximum surgical effort should always be pursued.

Among the multiquadrant surgical procedures usually required, bowel resections are the most common and the most frequently associated with severe postoperative complications.^6–9^

Albeit rare, anastomosis leakage (AL) is a life-threatening condition; its incidence varies widely in OC literature, ranging from 2.7 to 16.9%.^7,10–19^ AL is associated with prolonged hospitalization and increased time to chemotherapy, with negative impact on overall survival (OS).^6,7,10–15^

Compared with the colorectal cancer literature, there is a paucity of data on risk factors for AL during OC surgery, with few showing statistical significance, such as previous pelvic irradiation, poor nutritional status, and distance of anastomosis from anal verge.^6,17^

Recently, a multicenter retrospective study identified the at surgery, additional small bowel resections, and handsewn anastomosis as other possible risk factors.^20^

The aim of this study is to assess the AL rate in patients receiving colorectal resection and anastomosis during primary surgery (PDS or IDS) for OC, through a standardized surgical technique, in a high-volume tertiary cancer center specialized on OC treatment. In addition, we evaluated several possible pre/intra- and postoperative risk factors for AL.

## Methods

This study is a single-center retrospective, observational cohort study.

All pre-, intra-, and postoperative characteristics of patients who underwent primary surgery (PDS or IDS) for advanced epithelial OC at the Department of Gynecologic Oncology, Fondazione Policlinico Universitario A. Gemelli IRCCS, between December 2011 and October 2019, were collected. All demographic and surgical variables were retrieved from our prospective electronic database (REDCAP).

Informed consent was obtained from all women for their data to be registered and analyzed for scientific purposes. The trial was approved by our Ethics Committee (protocol ID no. 3304).

### Patients Clinical and Surgical Data

The enrolled population included all patients with histological diagnosis of epithelial ovarian, fallopian, or peritoneal cancer (FIGO stage IIIB–IVB) who underwent rectosigmoid resection and anastomosis with curative intent. All patients received preoperative mechanical bowel preparation.

Patients with end-colostomy or end-ileostomy were excluded from the study.

Several system scores, clinical and surgical variables, helpful in predicting operative and postoperative risk, were used to classify patients’ risk.

Patients with American Society of Anesthesiologists (ASA) score > 2, Eastern Cooperative Oncology Group-Performance Status (ECOG-PS) ≥ 2, and Age-Adjusted Charlson Comorbidity Index (ACCI) score > 2 were considered at high risk of postoperative complications.

Preoperative albumin level below 30 mg/dL and preoperative hemoglobin values below 10.0 g/dL were indicative respectively of severely poor nutritional status and moderate to severe anemia.

Nutritional status was also assessed by body mass index (BMI) classification (divided into the following categories: underweight patients with BMI < 18 kg/m^2^, normal weight–overweight with BMI 18–30 kg/m^2^, and obese patients with BMI ≥ 30 kg/m^2^).

CA-125 value and Predictive Index Value (PIV) at initial diagnosis were considered as potential indicators of tumor burden. To identify the disease with the greatest tumor burden, the cutoffs were set to CA-125 ≥ 1000 U/mL and PIV ≥ 6.

A cutoff of 60 years was used, based on the median age of the study population. Suspicion of AL, suggested by clinical signs such as abdominal pain or distension, leukocytosis, fever, presence of gas, pus, or feces in the drains, the abdominal incision, or vagina was confirmed by computed tomography (CT) scan with rectal contrast enema and/or immediate relaparotomy.

AL was defined as communication between the intra- and extraluminal compartments due to a defect in the integrity of the intestinal wall originating from the staple line of the neorectal reservoir between the colon and rectum. Pelvic abscess adjacent to the anastomosis were also categorized as anastomotic leakage even if no communication could be demonstrated with the colonic lumen at the anastomosis.^21^

The Common Terminology Criteria for Adverse Events v3.0 (CTCAE) was used to classify intraoperative complications (CTCAE 0–1 versus ≥ 2). The Extended Clavien–Dindo classification of surgical complications was used for the grading definition of early complications.^22^

OS was calculated from date of primary diagnosis to date of death or last follow-up.

### Surgical Technique

All colorectal resections and anastomoses were performed by a dedicated team of general surgeon who routinely collaborate with our department. Regarding the resection technique, when possible, we tended to perform low ligation of the lower mesenteric artery, thus preserving the left colic artery. In some cases, selective ligation of the sigmoid arteries was performed, preserving the superior rectal artery to improve the vascularization of the rectal stump. In case of doubt regarding intestinal vascularity, we used a coarse evaluation of the pulsatile flow and/or a marginal artery section to the proximal colon.

The mobilization of the colon for the execution of tension-free anastomosis involved the following steps: The first surgical maneuver was mobilization of the splenic flexure through the development of the avascular plane between the Gerota’s fascia and the left Toldt’s fascia. Second, the mobilization continued by dissecting the pancreatic–colic ligament. The ligation and section of the mesenteric vessels (first sacrificing the inferior mesenteric vein and/or the sigmoid vessels up to the ligation and sectioning of the inferior mesenteric artery) were maneuvers considered when the tension at the level of the anastomosis was still considered excessive by the surgeon. Colorectal anastomosis was performed using a circular stapling device for either end-to-end or side-to-end anastomosis. An air leakage test was performed immediately after completion of anastomosis. In case of intraoperative leakage, additional overstitches were placed.

In rare cases where the tissues were damaged or excessive leakage was found at the intraoperative air leakage test, the anastomosis was repackaged.

The level of the anastomosis was considered medium–low if the distance from the anal verge was less than 10 cm as measured by the rigid probe. The cutoff was set at 10 cm as this was considered and assumed to be the average length of the “extraperitoneal” rectum (medium–low rectum).

Diverting ostomy was performed based on the clinical status of the patient before surgery (compromised clinical condition, judged mainly by ASA and EOCG scores), the surgical procedures performed (multiple bowel resection), specific characteristics of the anastomotic complex (such as the level of anastomosis, the quality of the tissues, and their vascularization), and in general the surgical complexity (also judged by operative time and need for intraoperative transfusions).

### Statistical Analysis

Considering that the leakage rate for OC ranges between 2.7% and 16.9%,^7,10–19^ a sample size of *N* = 515 patients was calculated to be appropriate to detect a 16.9% AL (conservative approach), with *α* = 0.05 and a margin of error of 3.23%.

Descriptive statistics were used to describe the surgical procedures, patients, and pathological characteristics.

Absolute frequency and percentage were adopted for qualitative variables, or median and interquartile range (IQR) for continuous variables. The normality of data was verified via the Kolmogorov–Smirnov test. Groups were compared using the Mann–Whitney *U* test for continuous variables and the Pearson *χ*^2^ test or Fisher exact test for categorical variables.

Binomial logistic regression was performed to ascertain the effects of independent variables, judged as possible risk factors by clinicians, on the occurrence of AL and on the risk of diverting ostomy.

Multivariable logistic regression analysis was performed using a backward stepwise (likelihood ratio) model. Only the significant variables were included in the multivariable model. *p*-Value ≤ 0.050 was considered statistically significant (two-tailed test).

Survival analysis was performed using the Kaplan–Meier method and the Cox regression models.

Statistical analyses were performed using SPSS version 24.0 (IBM, Armonk, New York, NY) software for Windows.

## Results

### Patient and Surgical Data

Five-hundred fifteen (515) patients [372 (72.2%) PDS and 143 (27.8%) IDS] were identified in the study period.

Clinical features of the study population are presented in Table 1.

**Table 1:** Clinical and pathological features of the study population

Variable	All *n* (%)
No. of cases	515
Age (years)	
<60	277 (53.8)
≥60	238 (46.2)
BMI (kg/m^2^)	
<18	25 (4.9)
18–29.9	425 (82.5)
≥30	65 (12.6)
ASA	
≤2	493 (95.7)
>2	22 (4.3)
ECOG-PS	
0–1	495 (96.1)
2	20 (3.9)
AACCI	
0–2	337 (65.4)
>2	178 (34.6)
Smokers	
No	386 (79.6)
Yes	99 (20.4)
NA	30
PIV at primary diagnosis	
≤6	287 (62.3)
≥8	174 (37.7)
NA^a^	54
Hb pre surgery	
<10.0 g/dL	52 (10.1)
≥10.0 g/dL	463 (89.9)
Preoperative albumin value (mg/dL)	
≤30.0 mg/dL	46 (10.8)
>30.0 mg/dL	380 (89.2)
NA	89
CA-125^b^	
<1000 U/mL	254 (55.0)
≥1000 U/mL	208 (45.0)
NA	53
Ascites	
<500 cc	286 (55.5)
≥500 cc	229 (44.5)
Surgical timing	
PDS	372 (72.2)
IDS	143 (27.8)
Number of cycles^c,d^	4.00 (3.00–5.00)
Time from CHT to surgery (days)^c,d^	40.00 (34.00–46.50)
Bevacizumab^d^	
Yes	40 (28.0)
No	103 (72.0)

^a^Patients referred to NACT by external centers and who therefore did not undergo diagnostic laparoscopy

^b^Value at first diagnosis

^c^Median (range)

^d^Only IDS patients

*BMI* body mass index, *ASA* American Society of Anesthesiologists, *ECOG-PS* Eastern Cooperative Oncology Group-Performance Status, *AACCI* age-adjusted Charlson Comorbidity Index, *NA* not available, *PIV* Predictive Index Value, *Hb* hemoglobin, *PDS* primary debulking surgery, *IDS* interval debulking surgery, *CHT* chemotherapy

The study population was almost equally divided between women under the age of 60 years and (53.8%) and older women (46.2%). Of the study population, 12.6% was obese (BMI ≥ 30 kg/m^2^) while 4.9% was underweight (BMI < 18 kg/m^2^). Forty-six patients (10.8%) had preoperative serum albumin values below 30 mg/dl, while 52 (10.1%) had preoperative hemoglobin values below 10.0 g/dl. Almost all patients had an American Society of Anesthesiologists (ASA) score of 2 or less (95.7%), and an Eastern Cooperative Oncology Group-Performance Status (ECOG-PS) equal to or less than 1 (96.1%).

Surgical characteristics of the studied population are presented in Table 2.

**TABLE 2:** Surgical characteristics of study population

Variable	All *n* (%)
No. of cases	515
SCS groups	
1–2	155 (30.1)
3	360 (69.9)
Bowel resection	
1	400 (77.7)
>1	115 (22.3)
Splenectomy	
No	352 (68.3)
Yes	163 (31.7)
Hepatic resection	
No	478 (92.8)
Yes	37 (7.2)
Lymphadenectomy	
None	270 (52.4)
P elvic	49 (9.5)
Lumbo-aortic	196 (38.1)
Urological procedure	
No	505 (98.1)
Yes	10 (1.9)
Partial resection of the bladder	6 (60%)
Ureteral reimplantation	4 (40%)
Level of IMA section	
Section at the origin	128 (24.9)
Preservation of the left colic artery	387 (75.1)
Type of anastomosis	
End-to-end	395 (76.7)
Side-to-end	120 (23.3)
Hypogastric vessels section	
No	496 (96.3)
Yes	19 (3.7)
Distance of resection from anal verge	
<10 cm	128 (24.9)
≥10 cm	387 (75.1)
Protective ostomy	
None	285 (55.3)
Ileostomy/colostomy	230 (44.7)
Intraoperative complications	
CTCAE 0–1	494 (95.9)
CTCAE ≥ 2	21 (4.1)
HIPEC	
No	475 (92.2)
Yes	40 (7.8)
EBL	
≤500 cc	199 (38.6)
>500 cc	316 (61.4)
Intraoperative transfusions	
No	387 (75.1)
Yes	128 (24.9)
RT	
0	410 (79.6)
≤1 cm	84 (16.3)
>1 cm	21 (4.1)
Operative time (min)^a^	
Median (I–III quartile)	354.00 (278.00–443.00)
<300	169 (32.8)
≥300	346 (67.2)

^a^ Operative time includes HIPEC infusion time, when performed (40 cases)

*SCS* Surgical Complexity Score, *IMA* inferior mesenteric artery, *CTCAE* Common Terminology Criteria for Adverse Events, *HIPEC* hyperthermic intraperitoneal chemotherapy, *EBL* estimated blood loss, *RT* residual tumor

Optimal debulking was achieved in 95.9% of the enlisted population (CGR, 79.6%; RT (residual tumor) ≤ 1, 16.3%) while RT > 1 cm was left in the remaining population (4.1%). The Surgical Complexity Score (SCS) was generally elevated, with 69.9% of patients belonging to group 3 (high complexity score group).^23^ Regarding the specific characteristics of the rectosigmoid resection, high resection was usually performed (75.1%), with left colic artery sparing in 75.1% of cases. Based on patients’ clinical characteristics, surgical complexity, and the surgeon’s decision, protective ostomy (ileostomy or colostomy) was performed in 230 patients (44.7% of cases). Twenty-one patients (4.1%) had intraoperative complications of grade 2–4. Estimated blood loss (EBL) above 500 ml was recorded in 61.4% of cases, while intraoperative transfusions were required in 24.9% of patients.

Postoperative features are described in Table 3.

**Table 3:** Postoperative characteristics

Variables	All *n* (%)
No. of cases	515
Hospital stay (days)	8 (5–13)*
Postoperative complications^a^	267 (51.8)
G III–V	100 (19.4)
Postoperative anemia^a^	231 (44.9)
G III–V	28 (5.4)
Abdominal collection^a^	55 (10.7)
G III–V	26 (5.0)
Pancreatic fistula^a,b^	20 (12.3)
G III–V	8 (4.9)
Anastomotic leakage^a^	15 (2.9)
G III–V	12 (2.3)
G II	3 (20.0)
G III	5 (33.3)
G IV	4 (26.7)
G V	3 (20.0)
Diagnosis of anastomotic leakage (days)	5 (4–10.5)*
Time to chemotherapy (days)^d^	39 (33–47)*
Early postoperative mortality rate	14 (2.7)
Ostomy reversal^c^	
No	77 (33.5)
Yes	153 (66.5)
Time to ostomy reversal (months)	7 (3–37)*

^*^Median (I–III interquartile)

^a^Classified using the extended Clavien–Dindo classification of surgical complications

^b^Calculated on 163 patients subjected to splenectomy +/– distal pancreatectomy

^c^Calculated on 230 patients subjected to ostomy

^d^17/515 patients did not start postoperative chemotherapy due to postoperative complications (3.3%)

*G* grade, *NA* not available, *FUP* follow-up

The median hospital stay was 8 days, with 100 patients (19.4%) suffering severe postoperative complications (grade III–V). Fourteen patients (2.7%) died within 90 days from surgery.

Median time to chemotherapy was 39 days, but 17 patients (3.3%) could not start or resume chemotherapy due to postoperative complications (deterioration of physical condition or death).

Of the 230 patients receiving protective ostomy, 153 (66.5%) underwent ostomy reversal, with a median time of 7 months. The remaining 77 had no reversal due to disease recurrence or refusal to undergo further surgery.

### Study Protocol Results

The registered AL rate was 2.9% (15 out of 515 patients) (Table 3).

Diagnosis of AL was generally made on postoperative day 5 (interquartile range (IQR): 4–10.5 days).

Seven of the 15 patients with AL (46.7%) had diverting ostomy during first surgery; 3 could be treated conservatively with drainage plus broad-spectrum antibiotic therapy, while the rest underwent reoperation with resection of the anastomotic complex and colostomy according to Hartmann procedures. All eight patients with AL and without diverting ostomy required surgical reintervention (six were treated with the Hartmann procedure, and two received ileostomy) (Supplementary Table S1). The following variables showed a statistical significant association with AL on univariate analysis (Table 4): age at surgery ≥ 60 years (odds ratio (OR): 3.307, 95% confidence interval (CI): 1.039–10.527, *p* = 0.043), body mass index (BMI) < 18 kg/m^2^ (OR: 16.461, 95% CI: 5.191–52.196, *p* < 0.001), preoperative albumin value < 30 mg/dL (OR: 5.671, 95% CI: 1.772–18.143, *p* = 0.003), pelvic lymph node resection (OR: 4.269, 95% CI: 1.297–14.051, *p* = 0.017), section at the origin of the inferior mesenteric artery (IMA) (OR: 9.002, 95% CI: 2.814–28.801, *p* < 0.001), hypogastric vessels section (OR: 11.758, 95% CI: 3.354–41.220, *p* < 0.001), distance of the anastomosis from the anal verge < 10 cm (OR: 21.761, 95% CI: 4.840–97.838, *p* < 0.001), intraoperative transfusions (OR: 3.619, 95% CI: 1.286–10.187, *p* = 0.015), and postoperative anemia (OR: 5.132, 95% CI: 1.431–18.412, *p* = 0.012).

**TABLE 4:** Risk factors for anastomotic leakage: univariate and multivariate analyses

Variable	Univariate analysis		Multivariate analysis	
	OR (95% CI)	*p*-Value	OR	*p*-Value
Age (years)				
<60	Reference		Reference	
≥60	**3.307 (1.039–10.527)**	**0.043**	5.773 (0.785–41.887)	0.085
BMI (kg/m^2^)				
18–29.9	Reference		Reference	
<18	**16.461 (5.191–52.196)**	**< 0.001**	**19.621 (3.394–113.447)**	**0.001**
≥30	0.814 (0.100–6.621)	0.848	0.218 (0.004–10.735)	0.444
ECOG			–	–
0–1	Reference			
2	1.808 (0.226–14.473)	0.557		
ACCI			–	–
0–2	Reference			
>2	2.218 (0.791–6.221)	0.130		
Preoperative albumin value (mg/dL)				
≥30.0	Reference		Reference	
<30.0	**5.671 (1.772–18.143)**	**0.003**	**20.639 (2.345 –181.669)**	**0.006**
Type of surgery			–	–
IDS	Reference			
PDS	5.553 (0.723–42.623)	0.099		
Ascites			–	–
<500	Reference			
≥500	0.828 (0.290–2.362)	0.724		
PIV at first diagnosis			–	–
<6	Reference			
≥8	0.590 (0.185–1.884)	0.373		
SCS			–	–
1–2	Reference			
3	0.857 (0.288–2.550)	0.782		
No. of bowel resections			–	–
1	Reference			
≥2	1.274 (0.398–4.080)	0.683		
Splenectomy			–	–
No	Reference			
Yes	1.456 (0.510–4.163)	0.483		
Hepatic resection			–	–
No	Reference			
Yes	3.426 (0.963–12.727)	0.066		
Lymph node resection				
None	Reference		Reference	
Pelvic	**4.269 (1.297–14.051)**	**0.017**	5.274 (0.878–31.700)	0.069
Lumbo-aortic	0.584 (0.149–2.287)	0.440	0.453 (0.061–3.354)	0.438
Level of IMA section				
Preservation of the left colic artery	Reference		Reference	
Section at the origin	**9.002 (2.814–28.801)**	**< 0.001**	**10.732 (1.862–61.846)**	**0.008**
Hypogastric vessels section				
No	Reference		Reference	
Yes	**11.758 (3.354–41.220)**	**< 0.001**	6.412 (0.427 –96.373)	0.179
Distance of anastomosis from anal verge				
≥10 cm (high)	Reference		Reference	
<10 cm (mid–low)	**21.761 (4.840–97.838)**	**< 0.001**	**14.673 (2.340–92.006)**	**0.004**
Protective ostomy			–	–
None	Reference			
Ileostomy/colostomy	1.087 (0.388–3.043)	0.874		
HIPEC			–	–
No	Reference			
Yes	0.844 (0.108–6.591)	0.872		
Intraoperative complications			–	–
CTCAE 0–1	Reference			
CTCAE ≥ 2	3.895 (0.820–18.492)	0.087		
Operative time (min)			–	–
≤300	Reference			
>300	1.988 (0.533–7.141)	0.292		
EBL			–	–
≤500 mL	Reference			
>500 mL	4.226 (0.943–18.930)	0.060		
Intraoperative transfusions				
No	Reference		Reference	
Yes	**3.619 (1.286–10.187)**	**0.015**	0.869 (0.132–5.744)	0.884
RT			–	–
0	Reference			
0.1–1 cm	2.000 (0.612–6.536)	0.251		
>1 cm	2.000 (0.244–16.400)	0.518		
Postoperative anemia (Hb ≤ 8.0 g/dL)				
No	Reference		Reference	
Yes	**5.132 (1.431–18.412)**	**0.012**	4.223 (0.641 –27.816)	0.134

Variables included in multivariate analysis: age, BMI, preoperative albumin value, lymph node resection**,** level of IMA section, hypogastric vessels section**,** distance of anastomosis from anal verge, intraoperative transfusions, and postoperative anemia

*OR* odds ratio, *CI* confidence interval, *BMI* body mass index, *ECOG-P* Eastern Cooperative Oncology Group-Performance Status, *AACCI* age-adjusted Charlson Comorbidity Index, *PDS* primary debulking surgery, *IDS* interval debulking surgery, *PIV* Predictive Index Value, *SCS* Surgical Complexity Score, *IMA* inferior mesenteric artery, *HIPEC* hyperthermic intraperitoneal chemotherapy, *CTCAE* Common Terminology Criteria for Adverse Events, *EBL* estimated blood loss, *RT* residual tumor, *Hb* hemoglobin

Among the variables included in multivariate logistic regression analysis, only the following were identified as independent predictors of AL: BMI < 18 kg/m^2^ (OR: 19.621, 95% CI: 3.394–113.447, *p* = 0.001), preoperative albumin value < 30 mg/dL (OR: 20.639, 95% CI: 2.345–181.669, *p* = 0.006), section at the origin of the IMA (OR: 10.732, 95% CI: 1.862–61.846, *p* = 0.008), and distance of the anastomosis from the anal verge < 10 cm (OR: 14.673, 95% CI: 2.340–92.006, *p* = 0.004).

Supplementary Table S2 reports several pre- and intraoperative risk factors for diverting ostomy. Among all the analyzed variables, only the following showed increased risk on multivariate analysis: more than one bowel resection (OR: 5.412, 95% CI: 3.097–9.456, *p* < 0.001), medium–low anastomosis at less than 10 cm from the anal verge (OR: 2.414, 95% CI: 1.446–4.030, *p* = 0.001), operative time longer than 300 min (OR: 1.680, 95% CI: 1.023–2.758, *p* = 0.040), and need for intraoperative transfusions (OR: 1.688, , 95% CI: 1.031–2.763, *p* = 0.037).

Table 5 compares risk factors for diverting ostomy and anastomotic leak.

**TABLE 5:** Comparison of risk factors for diverting ostomy and anastomotic leak

Factors associated with diverting ostomy				Factors associated with AL			
Variable	OR	IC	*p*-Value	Variable	OR	IC	*p*-Value
No. of bowel resection ≥ 2	5.412	3.097–9.456	<0.001	BMI < 18 kg/m^2^	19.621	3.394–113.447	0.001
Distance of anastomosis from anal verge < 10 cm	2.414	1.446–4.030	0.001	Preoperative albumin level < 30 mg/dL	20.639	2.345–181.669	0.006
Operative time > 300 min	1.680	1.023–2.758	0.040	Section at origin of IMA	10.732	1.862–61.846	0.008
Intraoperative transfusions	1.688	1.031–2.763	0.037	Distance of anastomosis from anal verge < 10 cm	14.673	2.340–92.006	0.004

*AL* anastomotic leak, *BMI* body mass index, *IMA* inferior mesenteric artery

### Postoperative Features and Survival Analysis

Regarding the early postoperative course (Table 6), a significant difference among patients with and without AL was noted in length of hospital stay (8 versus 23.5 days, *p* = 0.001) and time to chemotherapy (38 versus 49.5 days, *p* = 0.013). Three patients with AL (20%) died from multiorgan failure (MOF) (Table 6) within 90 days versus 2.2% in women without AL (2.2% versus 20%, *p* = 0.001).

**Table 6:** Postoperative characteristics of patients with and without anastomotic leakage

Variable	No AL *n* (%)	AL *n* (%)	*p*-Value
No. of cases	500	15	
Hospital stay (days)	8 (5–13)^‡^	23.5 (11.5–34.5) ^‡^	0.001*
Severe early postoperative complication	87 (17.4)	13 (86.7)	**0.001**^**γ**^
Time to chemotherapy (days)	38 (32.25–46) ^‡^	49.5 (41–62.75) ^‡^	0.013*
Patients who could not start chemotherapy	14 (2.8%)	3 (20%)	**0.002**^**γ**^
Early postoperative mortality rate (90 days)	11 (2.2%)	3 (20%)	**0.001**^**γ**^

^‡^ Median (I–III interquartile)

^*^ Mann–Whitney *U*-test

^γ^ Pearson chi-squared test

*AL* anastomotic leakage

Median OS was 28 months in the AL group versus 50 months in the no-AL group, although the difference was not statistically significant (HR 1.767, 95% CI 0.869–3.594, *p* = 0.116) (Supplementary Fig. S1).

Supplementary Table S3 reports ostomy-related complications. We detected an overall rate of ostomy-related complications of 33.9% (78 out of 230 patients), of which only 16 were grade ≥ 3 (7%).

The most frequently reported complication was dehydration (23.9%), with most patients requiring i.v. fluid therapy (22.2%), and four patients (1.7%) underwent early ostomy reversal.

Forty-five patients (19.6%) were admitted to the emergency department due to ostomy-related complications, but as manty as 87 patients (37.8%) reported difficulties in ostomy management and impaired quality of life.

## Discussion

### Summary of Main Results

AL was confirmed as a rare postoperative complication in OC surgery, with only 15 reported cases in the entire population of 515 patients (2.9%).

Of the ALs, 80% were classified as “severe” since they required reintervention, and three patients died during post-operative course, leading to a significant increase in early postoperative mortality rate. Generally, patients with AL had statistically longer hospital stay and prolonged time to start of chemotherapy.

Shorter median OS was observed in patients with AL versus no AL (hazard ratio (HR) 1.767, 95% CI 0.869–3.594, *p* = 0.116) but the difference was not statistically significant. Therefore, AL could not be confirmed as a negative prognostic factor for OS.

Concerning variables that could increase the AL risk, the state of cachexia (demonstrated by low BMI and low preoperative albumin value) and specific characteristics of the colorectal anastomosis (ligation and section at the origin of the IMA and “mid–low level” anastomosis) resulted as relevant risk factors.

### Results in the Context of Published Literature

Several studies have shown how the consequences of AL can have a profound and negative impact on patients’ postoperative course, resulting in prolonged hospital stay, reduced probability of starting chemotherapy, and increased time to start of chemotherapy.^7,10–15^

Furthermore, some authors have recognized it as a significant negative prognostic factor in terms of 90-day mortality and OS, although these latter data did not reach statistical significance in our series.^15,24^

However, the identification of the population at greatest risk for AL, and proven strategies to prevent it, are still lacking today. Patients with OC represent a particular population since they usually present with critical clinical conditions and malnourishment, due to abundant ascitic fluid and widespread carcinomatosis.^25–27^ Moreover, given the predominantly peritoneal spread of OC, resections are usually higher than those performed for rectal cancer, and as suggested by Richardson et al.,^17^ this may explain the slightly lower rate of AL in OC patients.

When looking specifically at the OC literature, the studies appear inhomogeneous in assessing AL risk factors.^7,12,15^ This could be due both to its rarity and to the heterogeneity of the surgical technique used to perform the intestinal resection and anastomosis.^10–17,24^

According to literature data, we confirmed the low preoperative albumin value (≤ 30 mg/dL)^16,17,20,28–30^ and, in addition, identified low BMI (< 18 kg/m^2^) as independent preoperative risk factors for AL.

Among intraoperative variables, in agreement with several other studies, we confirmed medium–low level of anastomosis as a relevant risk factor of AL.^16,17,20,28^ The hypotheses proposed to explain this association are various, such as the major surgical difficulty when performing an anastomosis in the narrower and deeper portion of the pelvis, with greater tissue trauma, increased tension, and lower blood flow.^31^ In this context, section of the IMA at its origin was another important negative predictor identified, suggesting that a reduced blood supply could hinder correct healing of the anastomosis. This hypothesis was also suggested by Son et al.,^32^ who demonstrated reduced incidence of AL in patients with preservation of superior rectal artery.

As anticipated, conflicting data are reported on the efficacy of ostomy as a strategy to prevent AL,^33–44^ although a broad consensus exists on its role in preventing the catastrophic consequences of AL, with a reduction in morbidity and mortality.^34,36–38,40,45–48^

In our series, diverting ostomy did not prove to be a protective factor for AL, but it allowed conservative treatment in three patients who received it, which means a number needed to treat of 91 (91 ostomy to conservatively treat 1 AL). Another key to understanding these results could be that we correctly identify only 46.7% (7/15) of high-risk AL patients, in which we performed a protective ileostomy. Nevertheless, 57.1% (4/7) of these patients required a Hartmann procedure because of complete or nearly complete detachment of the anastomosis. These findings may indicate that there is a very small group of patients with an extremely increased risk of AL, where probably the best choice should be to perform a Hartmann procedure directly.

### Strengths and Weaknesses

The greatest bias in this study certainly lies in its retrospective nature, and we are aware that the limited number of AL events makes the risk factor analysis less reliable. Furthermore, this prevented an evaluation of interesting surgical maneuvers for the reduction of AL, such as techniques for mobilization of the descending colon to obtain an anastomosis as vascularized and tension free as possible. However, this is currently the largest monocentric study to specifically report the rate of AL and its main risk factors for patients with advanced OC undergoing PDS or IDS. Moreover, all the resections and anastomoses were performed by a dedicated team of general surgeons who routinely collaborate with gynecologic oncologists, and all the procedures were performed using the same surgical technique, further reducing the inherent limitations of a retrospective surgical study.

### Implications for Practice and Future Research

The findings of our study totally support the hypothesis that cancer cachexia, malnutrition, and chronic inflammatory activity (documented by low serum albumin value)^10,49^ are the most predictive preoperative factors of surgical morbidity, and strongly influence the postoperative course of OC patients,^50,51^ also increasing the incidence of AL. Therefore, increasing attention must be paid to preoperative optimization of the patient by developing programs that include physical exercise, and nutritional counseling with possible protein integration.^51,53^ Recently, in fact, a protocol was adopted in our department for preoperative optimization of the patient, providing both the association between mechanical bowel preparation and antibiotics, and careful nutritional assessment of the patient, protocols that were not active in the years considered in this study. Regarding the intraoperative details, data on the best colorectal resection and anastomosis in patients with advanced OC are lacking and the exact amount of blood flow needed to heal the intestinal wall remains to be determined.^31^ For this, it would be advisable to accurately tailor surgery according to tumor dissemination, to ensure maximal vascular supply to the anastomosis through preservation of the left colonic artery or, when possible, the upper rectal artery.^31,54,55^ For this purpose, intraoperative evaluation of anastomosis perfusion using indocyanine green could be an interesting topic for future studies.

Another currently open question is the usefulness of ostomy for AL prevention. We are aware of the high rate of ostomies performed, which we could explain with the high rate of optimal cytoreduction achieved (95.9% of cases, with 79.6% of CGR) and the high surgical complexity of the operations performed. In fact, in these cases, the surgeon, in an attempt to minimize the incidence and severity of postoperative complications, could be reassured by the diverting ostomy, although our results show that this did not prevent the onset of AL and allowed conservative treatment in a relatively low number of cases (NNT 91). In support of this, with the exception of the distance of the anastomosis from the anal verge, risk factors for ostomy and those for AL differed for the most part (Table 5). This means that the decision-making process that leads the surgeon to perform a protective ostomy is quite heterogeneous and based on personal experience and surgeon’s feelings, rather than actual risk factors for AL. However, considering the nonnegligible complication rate directly related to the ostomy itself, the nonreversal rate of 33.5%, and its negative psychological consequences,^56,57^ further studies are needed to better define the population that could really benefit from it and to make intraoperative decision-making as objective as possible

## Supplementary Information

Below is the link to the electronic supplementary material.

Supplementary file1 (DOCX 1967 kb)
