# Supplementary material for: Risk Factors for Anastomotic Leakage in Advanced Ovarian Cancer Surgery: A Large Single-Center Experience
Source: Ann Surg Oncol. 2022 Apr 18;29(8):4791–802. doi: 10.1245/s10434-022-11686-y (PMC9246984; doi:10.1245/s10434-022-11686-y)

**Table 1S** Surgical and post-operative details of patients with anastomotic leak

|  | **Diverting ostomy**  **n (%)** | **No Diverting Ostomy**  **n (%)** |
| --- | --- | --- |
| **Patients with AL** | 7 (46.7) | 8 (53.3) |
| **Managment of AL** |  |  |
| Conservative managment | 3 (42.9) | 0 (0.0) |
| Re-intervention | 4 (57.1) | 8 (100) |

AL anastomic leakage

**Table 2S** Risk factors for diverting ostomy: univariate and multivariate analysis

| **Variables** | **Univariate analysis** | | | **Multivariate analysis** | | |
| --- | --- | --- | --- | --- | --- | --- |
|  | **OR** | **IC** | **P value** | **OR** | **IC** | **P value** |
| **Age, years** |  |  |  |  |  |  |
| < 60 | Ref |  |  | - | - | - |
| ≥ 60 | 1.089 | 0.769-1.543 | 0.630 |  |  |  |
| **BMI, kg/m^2^** |  |  |  |  |  |  |
| 18-29.9 | Ref |  |  |  |  |  |
| < 18 | 1.010 | 0.448-2.275 | 0.982 | - | - | - |
| ≥ 30 | 1.325 | 0.786-2.235 | 0.291 |  |  |  |
| **ECOG** |  |  |  |  |  |  |
| 0-1 | Ref |  |  | - | - | - |
| 2 | 1.014 | 0.413-2.491 | 0.975 |  |  |  |
| **ACCI** |  |  |  |  |  |  |
| 0 – 2 | Ref |  |  | - | - | - |
| >2 | 1.169 | 0.812-1.684 | 0.401 |  |  |  |
| **ASA** |  |  |  |  |  |  |
| 1-2 | Ref |  |  | Ref |  |  |
| 3 | **2.244** | **0.925-5.447** | **0.074** | 1.983 | 0.683-5.757 | 0.208 |
| **Pre-operative albumin value (mg/dL)** |  |  |  |  |  |  |
| ≥ 30.0 | Ref |  |  | Ref |  |  |
| < 30.0 | **1.731** | **0.933-3.209** | **0.082** | 1.903 | 0.945-3.833 | 0.072 |
| **Type of surgery** |  |  |  |  |  |  |
| IDS | Ref |  |  | - | - | - |
| PDS | 0.957 | 0.649-1.409 | 0.822 |  |  |  |
| **Ascites** |  |  |  |  |  |  |
| < 500 | Ref |  |  | Ref |  |  |
| ≥ 500 | **1.363** | **0.960-1.934** | **0.083** | 1.367 | 0.872-2.142 | 0.173 |
| **PIV at first diagnosis** |  |  |  |  |  |  |
| < 6 | Ref |  |  | Ref |  |  |
| ≥ 8 | **1.970** | **1.361-2.852** | **<0.001** | 1.565 | 0.985-2.487 | 0.058 |
| **SCS** |  |  |  |  |  |  |
| 1-2 | Ref |  |  | Ref |  |  |
| 3 | **2.477** | **1.658-3.702** | **<0.001** | 1.572 | 0.943-2.620 | 0.083 |
| **N. of bowel resection** |  |  |  |  |  |  |
| 1 | Ref |  |  | Ref |  |  |
| ≥ 2 | **5.584** | **3.482-8.956** | **<0.001** | **5.412** | **3.097-9.456** | **<0.001** |
| **Level of IMA section** |  |  |  |  |  |  |
| Preservation of the left colic artery | Ref |  |  | - | - | - |
| Section at the origin | 0.913 | 0.610-1.366 | 0.657 |  |  |  |
| **Hypogastric vessels section** |  |  |  |  |  |  |
| No | Ref |  |  | Ref |  |  |
| Yes | **7.028** | **2.022-24.429** | **0.002** | 2.101 | 0.517-8.642 | 0.299 |
| **Distance of the anastomosis from the anal verge** |  |  |  |  |  |  |
| ≥ 10 cm (high) | Ref |  |  | Ref |  |  |
| <10 cm (mid-low) | **2.118** | **1.411-3.180** | **<0.001** | **2.414** | **1.446-4.030** | **0.001** |
| **HIPEC** |  |  |  |  |  |  |
| No | Ref |  |  | - | - | - |
| Yes | 1.262 | 0.662-2.407 | 0.480 |  |  |  |
| **Intraoperative complications** |  |  |  |  |  |  |
| CTCAE 0-1 | Ref |  |  | Ref |  |  |
| CTCAE ≥ 2 | **3.244** | **1.238-8.501** | **0.017** | 1.103 | 0.335-3.429 | 0.865 |
| **Operative Time** |  |  |  |  |  |  |
| ≤ 300 minutes | Ref |  |  | Ref |  |  |
| >300 minutes | **2.121** | **1.444-3.116** | **<0.001** | **1.680** | **1.023-2.758** | **0.040** |
| **EBL** |  |  |  |  |  |  |
| ≤ 500 mL | Ref |  |  | Ref |  |  |
| >500 mL | **2.027** | **1.404-2.924** | **<0.001** | 1.216 | 0.749-1.975 | 0.429 |
| **Intra-operative transfusions** |  |  |  |  |  |  |
| No | Ref |  |  | Ref |  |  |
| Yes | **2.633** | **1.744-3.976** | **<0.001** | **1.688** | **1.031-2.763** | **0.037** |

Variables included for the multivariate analysis: ASA, pre-operative albumin value, ascites, PI value, SCS, number of bowel resection, hypogastric vessels section, distance of the anastomosis from the anal verge, intra-operative complications, operative time, EBL, intra-operative transfusions

OR: odd ratio, CI: confidence interval, BMI: Body Mass Index, ECOG-P: Eastern Cooperative Oncology Group-Performance Status, AACCI: Age-Adjusted Charlson Comorbidity Index, ASA: American Society of Anesthesiologists, PDS: Primaty debulking surgery, IDS: Interval debulking surgery, PIV: Predictive Index Value, SCS: Surgical Complexity Score, IMA: inferior mesenteric artery, HIPEC: Hyperthermic intraperitoneal chemotherapy, CTCAE: Common Terminology Criteria for Adverse Events, EBL: estimated blood loss

**Table 3S** Ostomy related complications

| **Variables** | **230 n %** |
| --- | --- |
| **Ostomy related complications** All G | 78 (33.9) |
| G ≥III | 16 (7.0) |
| **Dehydration** All G | 55 (23.9) |
| G ≥III | 4 (1.7) |
| **Mucocutaneous detachment of the ostomy** All G | 9 (3.9) |
| G ≥III | 7 (1.3) |
| **Ostomy prolapse/stricture** All G | 14 (6.1) |
| G ≥III | 5 (2.2) |
| **A&E admission related to ostomy complications** | 45 (19.6) |
| **Difficulty in managing the stoma and**  **QoL’s impairment** | 87 (37.8) |

A&E accident and emergency, QoL quality of life

**Figure 1S** Kaplan-Meier plot for overall survival in patients with and without anastomotic leak


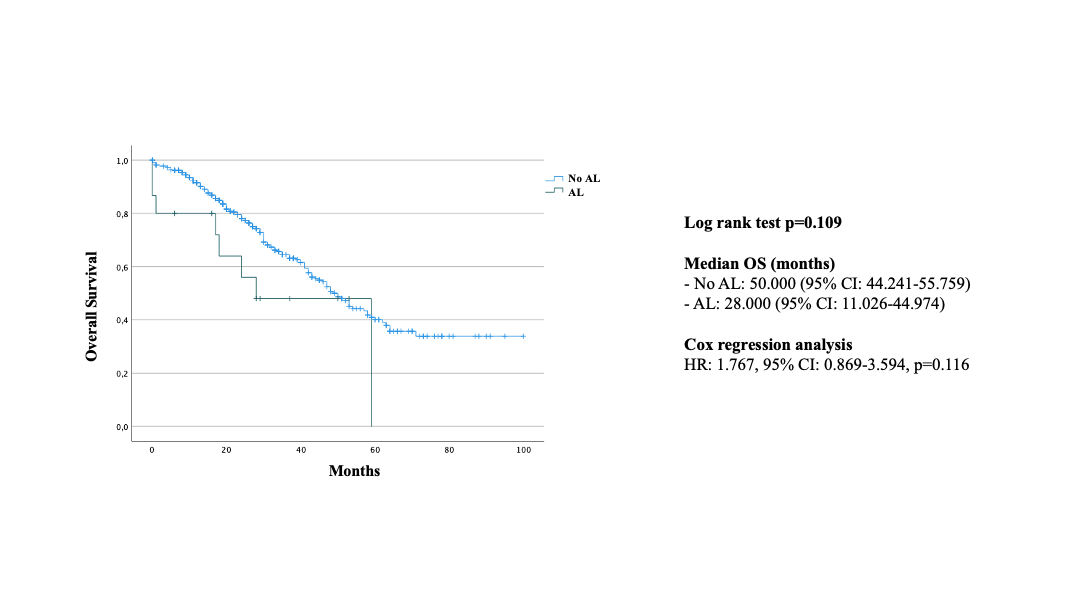

Supplement: Supplementary file 1 — Supplementary file1 (DOCX 1967 kb) [file 10434_2022_11686_MOESM1_ESM.docx]
